# Electrical impedance myography detects age-related muscle change in mice

**DOI:** 10.1371/journal.pone.0185614

**Published:** 2017-10-19

**Authors:** W. David Arnold, Rebecca S. Taylor, Jia Li, Janice A. Nagy, Benjamin Sanchez, Seward B. Rutkove

**Affiliations:** 1 Department of Neurology, The Ohio State University Wexner Medical Center, Columbus, Ohio, United States of America; 2 Department of Neurology, Beth Israel Deaconess Medical Center, Harvard Medical School, Boston, Massachusetts, United States of America; University of Sydney, AUSTRALIA

## Abstract

Loss of muscle mass and strength represents one of the most significant contributors to impaired function in older adults. Convenient and non-invasive biomarkers are needed that can readily identify and track age-related muscle change. Previous data has suggested electrical impedance myography (EIM) has the potential to serve in this capacity. In this study we investigated how changes in EIM compared with other standard measures of muscle structure and function in aged compared with young mice. A total of 19 male mice aged approximately 25 months and 19 male mice aged 3 months underwent surface multifrequency EIM of the right gastrocnemius muscle using standard methods. Fore and hind limb grip strength, sciatic compound muscle action potential amplitude, and in-situ force of the gastrocnemius were also measured; after sacrifice, gastrocnemius myofiber size was assessed using standard histology. Spearman correlation coefficients were calculated to investigate the association between EIM and muscle characteristics. EIM in aged animals demonstrated significantly lower 50 kHz impedance phase (p<0.001) and reactance (p<0.01) values as well as reduced multifrequency parameters. In contrast, absolute gastrocnemius muscle mass was no different between young and aged mice (p = 0.58) but was reduced in aged mice after normalization to body mass (p<0.001). Median myofiber size in the aged mice was not different from that of young mice (p = 0.72). Aged mice showed reduced muscle function on the basis of normalized fore limb (p<0.001) and normalized hind limb (p<0.001) grip strength, as well as normalized gastrocnemius twitch (p<0.001) and normalized maximal isometric force (p<0.001). Sciatic compound muscle action potential amplitude was reduced in aged mice (p<0.05). EIM parameters showed good correlation with reduced standard physiological and electrophysiological measures of muscle health. Our study suggests that EIM is sensitive to aged-related muscle change and may represent a convenient and valuable method of quantifying loss of muscle health.

## Introduction

Loss of muscle mass and strength, or sarcopenia, is an increasingly important public health problem that results as a consequence of aging in a large proportion of the older adults. There are no convenient, well-accepted diagnostic methods for the assessment of sarcopenia in at-risk individuals [1]. Sarcopenia was originally defined as loss of muscle mass [2, 3], but it has become increasingly apparent that loss of muscle strength is a more important indicator of muscle status during aging [4–7]. Thus, the definition of sarcopenia has changed to describe both loss of muscle strength, sometimes referred to as dynapenia, as well as loss of muscle mass itself [8, 9]. As such, methods for assessing sarcopenia and muscle status have also evolved over time and include measures of physical performance, imaging modalities to analyze muscle size and composition, quantitative strength testing, and molecular markers [1]. The concept of “muscle quality” has also been employed to describe loss of muscle strength out of proportion to loss of muscle mass that occurs in sarcopenia [5–7, 10–12]. Indeed, histological studies have demonstrated a number of alterations in aged muscle, including mild myofiber atrophy, increased connective tissue and fat deposition, and conversion of some muscle fibers from type 2 to type 1 [5–7, 13, 14]. Yet, a simple-to-use, non-invasive tool for sarcopenia assessment that provides information about muscle quality has not been identified.

Electrical impedance myography (EIM) is an emerging technology for the assessment of neuromuscular health that has already shown significant value in a variety of disorders affecting both muscle and nerve, including amyotrophic lateral sclerosis [15, 16], spinal muscular atrophy [17, 18], and Duchenne muscular dystrophy [19]. Whereas prominent pathological change, as occur in these disorders, is readily identified by EIM, the technique also appears sensitive to subtler disorders. For example, EIM alterations have been identified in disuse, both in an animal models and in humans [20, 21]; it has also been shown to be sensitive to the effects of microgravity on muscle [22]. Accordingly, we have also previously assessed EIM in older individuals and identified lower phase values compared with younger individuals [23]. In addition, 4 healthy older adults were also studied longitudinally, and reductions in EIM values were identified [23]. One additional study also identified changes in EIM values in paraspinal muscles of older individuals, consistent with sarcopenic change [24]. However, in these studies the underlying histological and functional status of the muscle and its association with EIM values were not investigated. Therefore, in this study, we evaluated EIM in aged and young mice to identify the technique’s relationship to standard functional and histological measures of muscle condition.

## Methods

### Ethics statement and experimental animals

This study was carried out in strict accordance with the recommendations in the *Guide for the Care and Use of Laboratory Animals* of the National Institutes of Health. Our animal protocol was approved by the Beth Israel Deaconess Medical Center Institutional Animal Care and Use Committee (IACUC) (Protocol Number 087–2016), and all efforts were made to minimize any animal suffering. C57BL/6 male mice were obtained from the National Institutes on Aging Animal Program at approximately 3 and 25 months (n = 19 per group). Animals were allowed to acclimate at least 72 hours prior to testing and were fed a standard diet *ad libitum*. Impedance and *in situ* force experiments were performed under 1–2% inhaled isoflurane anesthesia. At the conclusion of all studies, the animals were euthanized with carbon dioxide.

### Body mass and grip strength assessment

Animals were weighed with an analytical balance. Fore limb and hind limb grip strength were measured in grams by a grip strength meter with a sensor range of 0–1000 grams and accuracy of 0.25% of the full scale with standard pull bar (CAT #1027CSM, Columbus Instruments, Columbus, OH). For both, the animal was first allowed to grip the pull bar; the investigator, grasping the lower back of the animal then pulled it away from the bar until the mouse lost its grip. The maximum force recorded out of 5 trials was recorded. This test was performed separately on the fore and hind limbs.

### Animal preparation for EIM/CMAP and *in situ* force

Impedance and *in situ* force experiments were performed under 1–2% inhaled isoflurane anesthesia delivered by nose cone with body and muscle temperature being maintained by a heating pad (37°C). A depilatory agent was applied to the right hind limb to remove fur, and the skin was cleaned with 0.9% saline solution. Both legs were taped to the measuring surface at an approximately 45° angle extending out from the body in preparation for measurements.

### Compound muscle action potential (CMAP) amplitude

Sciatic CMAP amplitudes were recorded from the sciatic innervated triceps surae muscle of the right hindlimb using a TECA Synergy T2 EMG Monitor System (Viasys, Inc, Madison, WI) as previously described [25–27]. Briefly, the sciatic nerve was supramaximally stimulated at the sciatic notch. CMAP amplitude was recorded using two ring electrodes (Catalogue # 9013S0312, Natus Neurology, Middleton, Wisconsin, USA) placed on the proximal leg over the posterior and anterior compartment muscle groups (active electrode, E1) and on the mid-metatarsal region of the foot (reference electrode, E2). A ground electrode was placed on the left hind paw. CMAP amplitudes were measured peak-to-peak.

### Electrical impedance myography (EIM)

A fixed rigid 4-electrode impedance-measuring array was applied over the left gastrocnemius muscle. EIM measurements were performed with the EIM1103 System (Myolex, Inc, San Francisco, CA), which obtains impedance data at 41 frequencies from 1 kHz to 10 MHz as previously described [25, 28]. The 50 kHz phase, resistance, and reactance values were extracted from the frequency set as were the modeled multifrequency Cole parameters. The three Cole parameters of interest included the center frequency, *fc*, the dispersion coefficient alpha,*α*, and the cell density, *R0/Rinf* [29, 30]. The meaning of each of these is described in more detail below.

### *In situ* muscle physiology

After the CMAP and EIM measurements, a non-survival surgery was performed to expose the left gastrocnemius muscle and calcaneal tendon [31]. The soleus muscle and underlying fascia were dissected away from the calcaneal tendon; the tendon was then connected to a force lever arm (described in more detail below) and the leg stabilized by inserting a disposable monopolar needle (902-DMF37-S, Natus neurology, Middleton, Wisconsin, USA) through the knee joint. Twitch and tetanic force were recorded following stimulation of the sciatic nerve with 200 ms square pulses via insulated monopolar needles (F-E2M-48, Grass Technologies, Warwick, Rhode Island, USA). A high-speed servomotor-based apparatus (Model 305C, Aurora Scientific, Aurora, Ontario, Canada) was used to measure force output. The output signals from the lever system were interfaced to a PC-platform integrating a PXIe-8135 quad-core processor based embedded controller and a two-channel acquisition board PXI-4461 from National Instruments (Austin, Texas, USA). A custom program controlled the lever arm movement and output of a biphasic pulses current muscle stimulator (Model 701, Aurora Scientific). Stimulation current and resting tension were adjusted to maximize twitch force produced by a single stimulus pulse. Optimal length was measured with digital calipers as the distance between the knee and the calcaneal tendon. All subsequent isometric tetanic force data were collected at this stimulation current and resting tension. Isometric force frequency relationship was recorded after stimulation by a train of square wave stimuli at 100Hz, 110Hz, 120Hz, and 200Hz and the maximum isometric tetanic force was recorded. At the conclusion of all studies, the animals were euthanized with carbon dioxide.

### Muscle histology

Wet muscle mass of the excised gastrocnemius muscle was measured with a standard analytical balance. The right gastrocnemius was harvested from a subset of animals (9 mice aged 3-month and 9 mice aged 26-month-old animals) and placed in 10% formalin. Samples were then embedded in paraffin blocks, sectioned into 10 μm slices and stained with anti-collagen VI antibody (Abcam ab6588). Sections were subsequently imaged at 20x with a Zeiss AxioImager M1 epifluorescence microscope and fiber area was measured using Volocity^®^ software (PerkinElmer, Akron, Ohio, USA).

### Data analysis

Impedance data was processed with MATLAB (The Mathworks, Natick, MA) to extract the Cole parameters. Statistical analysis on the raw impedance values and Cole parameters was then performed using GraphPad Prism (GraphPad Software, Inc. La Jolla, CA); p < 0.05, two-tailed was considered significance for all tests. All mass, grip strength, force, and 50 kHz EIM data are reported as median with upper and lower 95% confidence intervals. We compared older and younger animals using Mann-Whitney tests. For correlation analyses, Spearman correlation coefficients were calculated. We analyzed the EIM parameters with two-way ANOVA to explore the effect of age and electrode orientation (longitudinal versus transverse orientation with the direction of the muscle fibers).

## Results

### Body mass, muscle mass, grip strength, and CMAP

**Fig 1:**
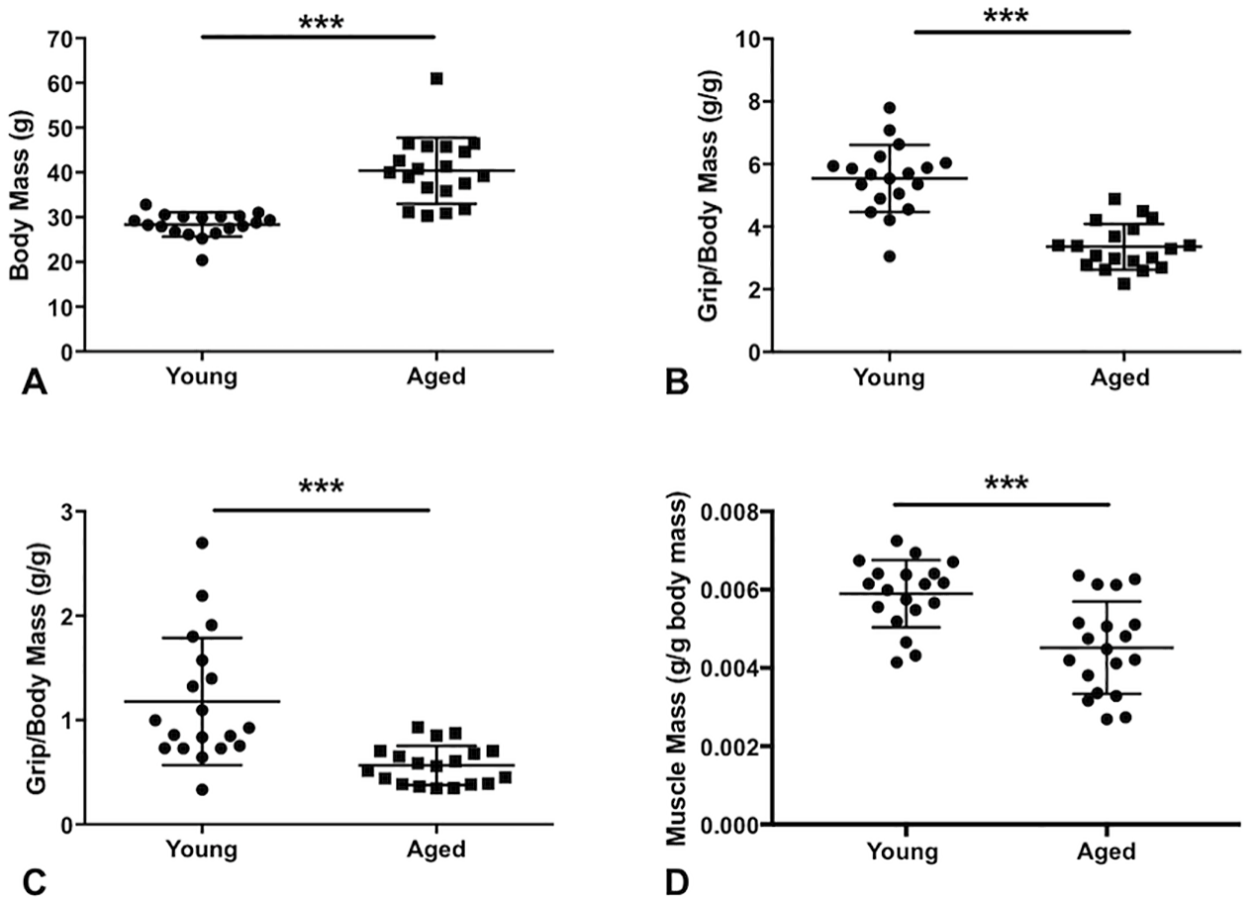
Mean body mass, muscle mass, and grip testing. Aged mice demonstrated increased body mass (A) reduced normalized fore limb grip (B), reduced normalized hind limb grip strength (C), and reduced normalized wet muscle mass (D) compared with young mice. ***, p<0.001. Data shown as mean ± standard deviation in Fig 1. Aged mice also demonstrated significant reduction in absolute forelimb (young = 160.0g, 138.0 to 174.0 g versus aged = 127.0g, 119.0 to 152.0g; p<0.001) and absolute hindlimb (young = 26.0g, 22.0 to 47.0g versus aged = 21.0g,16.0 to 29.0g; p<0.05) strength (not shown). In contrast, absolute gastrocnemius muscle mass was similar between aged and young mice (young = 0.167g, 0.137to 0.194g) versus aged mice = 0.186g, 0.156 to 0.194g); p = 0.58) (not shown).

Body mass, wet muscle mass, and grip strength are summarized in Fig 1 and Table 1. The aged mice were considerably heavier but absolute muscle mass remained virtually identical. Interestingly, despite having similar absolute muscle mass, both absolute and normalized fore limb and hind limb grip strength were significantly reduced in older animals. The fore limb strength data were more consistent, which is likely related to the relative technical ease of obtaining fore limb strength measurements as compared to hind limb. CMAP also demonstrated reduced amplitude in aged mice (52.7mV, 37.1 to 75.7mV) compared with young mice (62.2mv, 56.6 to 78.9mV) (p<0.05) (Data not shown in Fig 1).

**Table 1:** Body mass, wet muscle mass, and grip strength.

	Young	Aged	p value
Body mass (g)	28.79 (26.7, 30.15)	39.98 (35.87, 45.75)	<0.001
Absolute muscle mass (g)	0.167 (0.137, 0.194)	0.186 (0.156, 0.194)	0.58
Normalized muscle mass (g/g)	0.006142 (0.005484, 0.006411)	0.004484 (0.003358, 0.005148)	<0.001
Forelimb grip strength (g)	160.0 (138.0, 174.0)	127.0 (119.0, 152.0)	<0.001
Hindlimb grip strength (g)	26.0 (22.0, 47.0)	21.0 (16.0, 29.0)	<0.05
Normalized forelimb grip strength (g/g)	5.675 (4.894, 6.041)	3.293 (2.776, 3.927)	<0.001
Normalized hindlimb grip strength (g/g)	0.93 (0.73, 1.57)	0.56 (0.39, 0.70)	<0.001
CMAP amplitude (mV)	62.2 (56.6, 78.9)	52.7 (37.1, 75.7)	<0.05
Twitch force (mN)	607.0 (506.0, 787.0)	503.0 (410.0, 639.0)	<0.05
Isometric force (mN)	3120 (2846, 3352)	2860 (2445, 3198)	0.14
Optimal length (mm)	17.97 (17.17, 18.33)	18.16 (17.90, 18.45)	0.34
Normalized twitch (mN/g)	23.17 (16.37, 28.87)	12.98 (10.56, 15.70)	<0.001
Normalized maximum isometric (mN/g)	110.60 (102.10, 116.00)	73.21 (54.43, 87.78)	<0.001
Myofiber CSA (μm^2^)	1845 (1426, 2730)	1929 (1779, 2146)	0.72

For all group comparisons, a total of 19 young and 19 aged mice were analyzed excluding muscle fiber cross-sectional area (CSA), whereas young = 9 mice (1433 muscle fibers) and aged mice = 9 mice (893 muscle fibers). Grip strength and muscle force were normalized to body mass. Data shown as median with upper and lower 95% confidence intervals.

### Muscle physiology and muscle morphometrics

**Fig 2:**
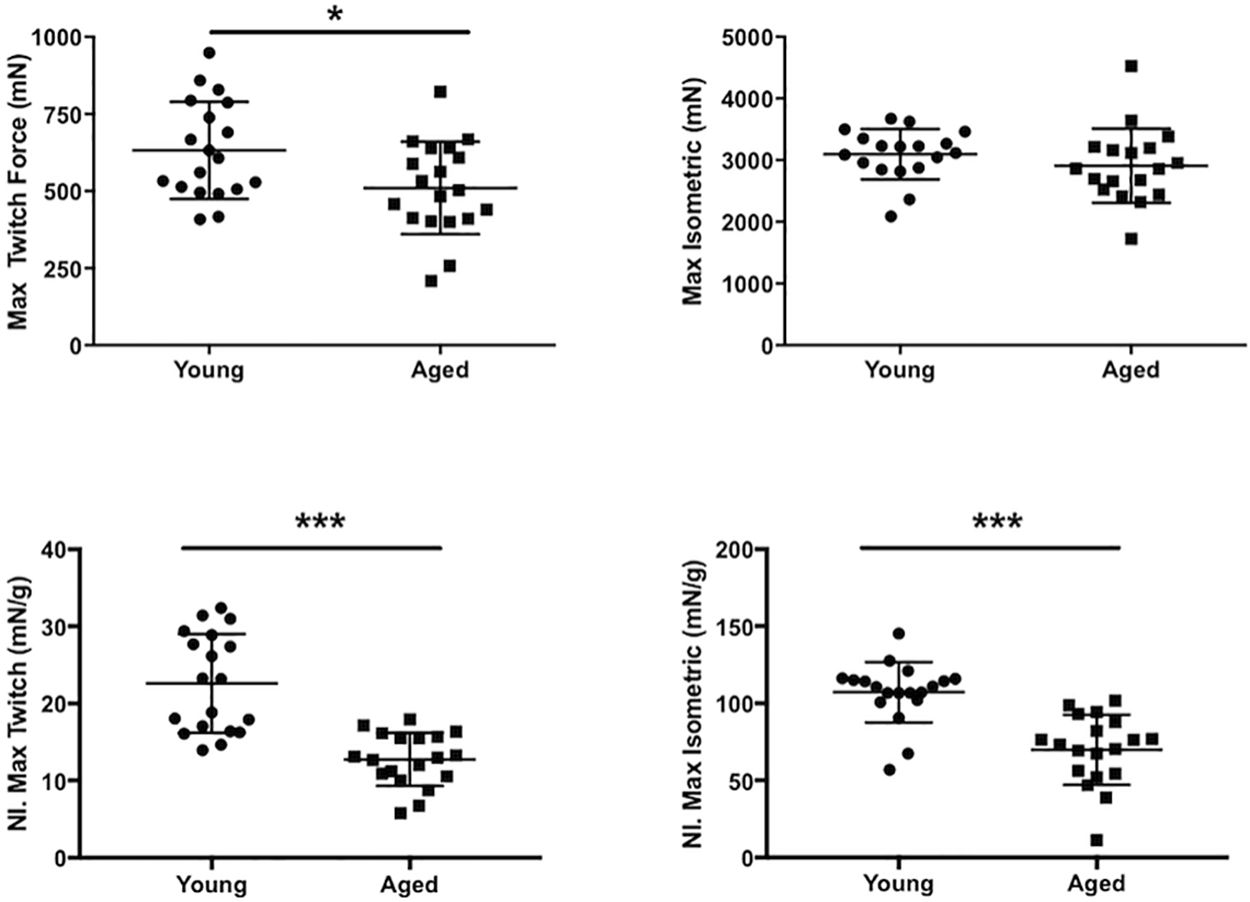
Muscle force testing. *In situ* testing of gastrocnemius muscle force demonstrates (A) reduced absolute twitch force in aged (n = 19) compared with young mice (n = 19) (p<0.05) and (B) similar absolute maximal tetanic isometric force measurements (p = 0.14). Both (C) normalized twitch (p<0.001) and (D) maximum isometric force are significantly reduced in the aged mice (p<0.001). Data shown as mean ± standard deviation.

**Fig 3:**
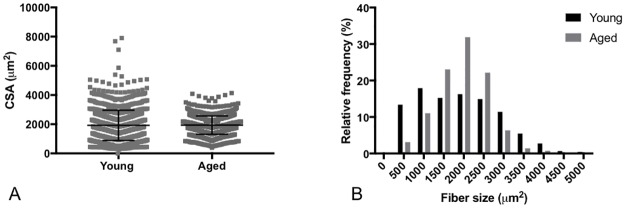
Muscle fiber cross-section area and frequency distribution. **(A)** There was no difference in myofiber CSA between the groups (p = 0.72). Young (n = 9 mice, 1433 muscle fibers measured) and aged mice (n = 9 mice, 893 muscle fibers measured). Data shown as mean ± standard deviation. (B) However, there is a decrease in muscle fiber size heterogeneity in the aged mice as compared to the young animals as evidenced by a narrowing of the frequency distribution for aged mice.

*In situ* muscle force data for the gastrocnemius is shown in Fig 2 and in Table 1. For the absolute values, twitch force shows a significant reduction in the aged mice whereas the maximal isometric force is not significantly different between the young and aged mice. When normalized to body mass, there is a significant reduction (p<0.001) in both twitch and maximal isometric force. Fig 3 shows the mean muscle fiber size and muscle fiber size distributions between groups. When comparing muscle fiber size between 9 young and 9 aged mice, cross sectional areas (CSA), grouped and averaged by each individual mouse, show no significant difference between groups (p = 0.72). The coefficient of variation (standard deviation/mean) of the CSA of the young mice (0.46 ± 0.08) was significantly increased (p<0.01) compared with that of aged mice (0.31± 0.08). This decrease in muscle fiber size heterogeneity in the aged mice can be seen in the narrowing of the frequency distribution histogram (Fig 3).

### Electrical impedance myography (EIM)

**Fig 4:**
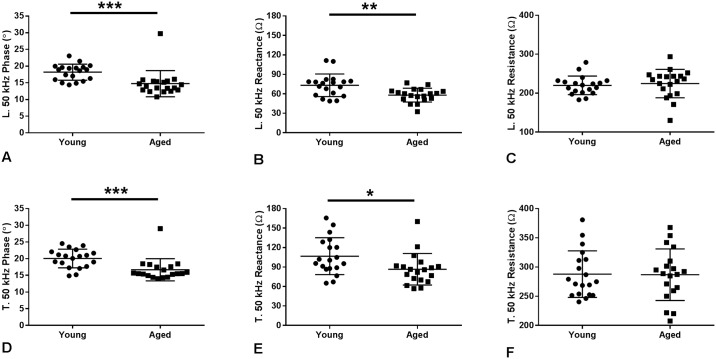
Electrical impedance myography (EIM) characteristics at 50 kHz. Longitudinal 50 kHz (A) phase (p<0.001) and (B) reactance (p<0.01) are reduced in aged mice, but (C) resistance is unchanged (p = 0.27). Similarly, transverse 50 kHz (D) phase (p<0.001) and (E) reactance (p<0.01) are reduced in aged mice but (F) resistance is unchanged (p = 0.84). Data shown as mean ± standard deviation.

**Fig 5:**
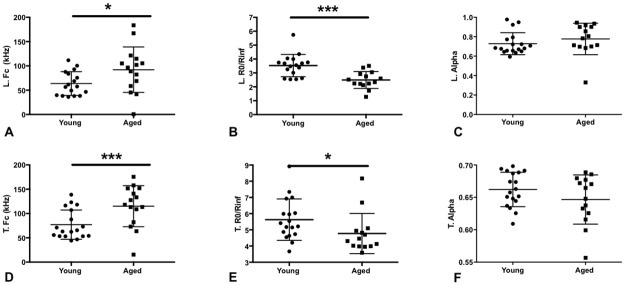
Electrical impedance cole parameters. A. Longitudinal *fc* is increased in aged mice (p<0.05). B. Longitudinal R0/Rinf is diminished in aged mice (p<0.001). C. In contrast, longitudinal alpha is unchanged (p = 0.084). D. Similarly, transverse *fc* is increased in aged mice (p<0.01). E. Transverse R0/Rinf is decreased in aged mice (p<0.05), and (F.) transverse alpha is again unchanged (p = 0.24). Data shown as mean ± standard deviation.

We evaluated single frequency impedance parameters at 50 kHz (Fig 4 and Table 2) that have previously been found to hold value in both human and animal studies. Cole multifrequency parameters, which attempt to provide insight into pathological changes in muscle on the basis of basic impedance theory, were also derived (Fig 5 and Table 2) [29]. The 50 kHz phase and reactance values both in the longitudinal and transverse directions showed major reductions, with differences in the phase being most pronounced. However, there was no significant change in the resistance values for either direction of measurement. Two-way ANOVA was also performed to examine the effects age and electrode orientation (longitudinal versus transverse) concurrently on EIM results. This analysis demonstrated significant effects on 50 kHz Phase by both age (p<0.001) and electrode orientation (p<0.05). Similarly, reactance was affected by both age (p<0.001) and electrode arrangement (p<0.001). In contrast, resistance was not affected by age (p>0.05) but is affected by electrode orientation (p<0.001). The Cole parameter of central frequency (*fc*) demonstrated significant differences between groups for both the longitudinal and transverse directions. The *fc*, which is inversely related to muscle fiber size, may be expected to be elevated in the older mice in which some subtle atrophy, perhaps not identified on histology, may be present. While the alpha value (a measure of cell-size variation within the animal’s muscle), failed to reveal a difference, the *R0/Rinf*, a measure of cell density, showed a significantly higher value for the younger mice, consistent with the presence of more cells/unit area. Stated another way, the reduced *R0/Rinf* in older animals could suggest an increase in non-cellular components (e.g., increased connective tissue or fat).

**Table 2:** 50 kHz and cole parameter results of longitudinal and transverse electrical impedance myography measurements of the left gastrocnemius.

		Young	Aged	p value
Longitudinal	50 kHz phase	18.9 (16.0, 19.6) n = 19	13.9 (12.8, 15.5) n = 19	<0.001
	50 kHz reactance	76.2 (57.9, 79.6) n = 19	60.2 (52.0, 64.3) n = 19	<0.01
	50 kHz resistance	220.6 (201.7, 232.0) n = 19	234.7 (198.7, 243.6) n = 19	0.27
Transverse	50 kHz phase	20.7 (17.7, 22.1) n = 19	15.5 (14.9, 17.6) n = 19	<0.001
	50 kHz reactance	100.6 (87.9, 128.7) n = 19	85.8 (70.1, 91.9) n = 19	<0.05
	50 kHz resistance	274.9 (252.6, 313.0) n = 19	286.9 (259.4, 310.2) n = 19	0.84
Longitudinal	fc (kHz)	57.5 (39.7, 83.8) n = 17	99.3 (58.7, 121.3) n = 14	<0.01
	R0/Rinf	3.56 (3.01, 3.76) n = 17	2.43 (2.12, 3.05) n = 14	<0.001
	alpha	0.682 (0.656, 0.772) n = 18	0.794 (0.697, 0.918) n = 14	0.084
Transverse	fc (kHz)	64.3 (53.4, 107.1) n = 18	122.9 (82.1 152.0) n = 14	<0.001
	R0/Rinf	5.31 (4.74, 6.03) n = 18	4.38 (3.98, 5.10) n = 14	<0.05
	alpha	0.6603 (0.644, 0.689) n = 18	0.656 (0.616, 0.680) n = 14	0.24

Phase shown in degrees, and reactance and resistance are shown in Ohms. Data presented as median with upper and lower 95% confidence intervals.

### Correlation of EIM with other measures

In addition to seeking to identify differences between the groups, we also sought to evaluate the relationship between the different impedance parameters and the more standard metrics we obtained. Table 3 shows the associations between longitudinal and transverse impedance values at 50 kHz combining both the young and aged mice. Both phase and reactance show good correlation with multiple measures of muscle function when measured in both longitudinal and transverse orientations. In contrast, 50 kHz transverse resistance correlated only with muscle mass and normalized tetanic force, and there were no significant correlations for longitudinal 50 kHz resistance.

**Table 3:** Correlations between 50 kHz electrical impedance myography parameters, grip strength normalized to body mass, muscle mass, normalized twitch force, and normalized tetanic force.

		Normalized forelimb grip	Normalized hindlimb grip	Muscle mass	Normalized twitch force	Normalized maximum force
Longitudinal	Phase	r = 0.68 p<0.001	r = 0.50 p<0.01	r = -0.39 p<0.05	r = 0.34 p<0.05	r = 0.42 p<0.05
	Reactance	r = 0.47 p<0.01	r = 0.38 p<0.05	r = -0.48 p<0.01	r = 0.39 p<0.05	r = 0.40 p<0.05
	Resistance	r = -0.18 p = 0.28	r = -0.13 p = 0.45	r = -0.14 p = 0.41	r = 0.08 p = 0.64	r = 0.14 p = 0.43
Transverse	Phase	r = 0.6 p<0.001	r = 0.46 p<0.01	r = -0.46 p<0.01	r = 0.37 p<0.05	r = 0.47 p<0.01
	Reactance	r = 0.34 p<0.05	r = 0.33 p<0.05	r = -0.48 p<0.01	r = 0.39 p<0.05	r = 0.61 p<0.001
	Resistance	r = -0.08 p = 0.66	r = 0.01 p = 0.96	r = -0.38 p<0.05	r = 0.27 p = 0.11	r = 0.46 p<0.01

A total of 38 mice (19 young and 19 aged) were included for each comparison excluding tetanic specific force (n = 36, 18 young and 18 aged mice). Spearman correlation coefficients (r) are shown.

## Discussion

This study demonstrates that EIM is sensitive to age-associated alterations in mouse muscle, supporting the potential of EIM to serve as a simple, non-invasive approach for assessing sarcopenic change in older men and women. A key finding in this study includes the fact that differences between old and young mice using standard assessment tools were fairly modest. This was particularly the case for comparison of myofiber CSA and absolute muscle mass, which suggested minimal differences between the young and aged mice. Other investigators have also observed that C57BL/6 mice show relatively mild changes in muscle at 24 months [32, 33]. Interestingly, despite the lack of difference in absolute gastrocnemius muscle mass and findings of similar muscle fiber size, the aged mice demonstrated reduced muscle function on grip and during *in situ* twitch force testing. Similarly, the electrophysiological status of muscle was altered in aged mice as revealed with reduced CMAP amplitude. The loss of CMAP amplitude, which represents the total electrophysiological response of a muscle or group of muscles following supramaximal stimulation of the innervating motor nerve, has also been noted previously in both prior clinical and preclinical studies of aging [34, 35]. Our findings of relatively preserved muscle mass but loss of force and CMAP amplitude are aligned with prior clinical studies demonstrating that loss of muscle strength is more rapid as compared with loss of lean mass in aging individuals [5–7]. Furthermore, this supports the fact that more recent consensus definitions of sarcopenia have been expanded to include not only loss of muscle mass but also reductions in muscle function [36].

What do the alterations in EIM values identified in this study suggest? First, it is important to point out that these impedance effects are unlikely to be related simply to increasing fat percentage of body composition with age. If that were the case, there would be an expectation of increasing resistance values (the impedance value most readily impacted by fat) in concert with the reductions in phase and reactance. However, resistance values were virtually identical in our studies, suggesting the phase and reactance are impacted by other factors. Generally, reactance, and consequently phase, would be expected to decrease if there were reductions in myofiber CSA. Similarly, the Cole parameter *fc* demonstrated a significant increase in the older animals, which would also be observed in the setting of myofiber atrophy. Interestingly while the 50 kHz reactance and phase are significantly reduced and *fc* is increased in the aged mice, standard morphometric analysis of muscle fiber CSA did not reveal any significant difference in muscle fiber size. The conflicting results between EIM and histology could reflect the fact that certain cell populations (e.g. severely atrophied myofibers due to axon dropout) were simply ignored when measuring fiber size using light microscopy. The reduction in *R0/Rinf* is also consistent with this possibility, since it suggests a decrease in cell density in the older mice (any small atrophic fibers behaving bioelectrically more as extracellular debris rather than actual cells). The Cole parameter alpha, α, is supposed to provide a measure of cell size distribution. In contrast to the sensitivity of most of the impedance parameters, α was not different between the young and old mice, despite the fact that the measured coefficient of variation in myofiber size revealed less variation in the aged mice.

Decreased muscle quality has been shown to be a good predictor of functional decline, morbidity, and mortality in aging individuals [7, 37]. Loss of muscle quality has been attributed to a number of underlying pathological and pathophysiological changes [38]. One potential cause includes the deposition of intramuscular fat with aging leading to impaired muscle contractility [12, 39–42]. Additionally, both the loss of motor neurons and the loss of motor unit connectivity at the neuromuscular junction have been implicated as demonstrated in a number of preclinical and clinical studies [43–50]. Regardless of the major factors that drive loss of muscle quality, EIM appears to be sensitive to these alterations as is apparent in the striking EIM differences between young and aged mice as well as EIM’s consistent correlations with muscle strength, mass, and function. In our view the relationship between obtained EIM values and the measured force generating capability of the muscle is the most compelling outcome from this study. Stated another way, it may be that EIM values can serve as a surrogate measures to actual force measurements and muscle quality. We have previously shown in both clinical and animal studies that, in a number of neuromuscular conditions, EIM parameters are related to force and function [15, 51–53]. Moreover, we have recently identified a relationship between lower extremity force and impedance values in healthy older adults [54]. This work provides further support for that relationship.

There are a number of limitations to this study that need to be highlighted. First, we obtained histological data only in a subset of animals due to a technical problem with our histological processing of the first sets of young and old animals studied. Second, it would have been very helpful to have saved additional tissue for analysis of connective tissue and fat deposition as we had done in some earlier work, which was not performed here. Increases in these components would have added insight into the observed impedance alterations [55]. Also, in this analysis, we only performed surface EIM measurements and no *ex vivo* studies, post-mortem. However, as we have seen in other work, the *ex vivo* measurements often simply reflect the *in vivo* findings, but are considerably more challenging to acquire [56].

This study raises a number of questions for future study. First, an additional dedicated study of the relationship between EIM alterations in aging muscle is needed. One next step may be to evaluate additional rodent models of accelerated aging [57]. Alternatively, investigation in human subjects, potentially including muscle biopsy to fully evaluate the tissue and relationship to impedance change, may be required. Regardless, the results presented here add to a growing body of literature supporting the potential application of EIM in the assessment of age-associated muscle deterioration.

## Supporting information

S1 File Raw data for Figs 1–5 and Tables 1–3. (XLSX)
